# High-Intensity Interval Training (HIIT) on Biological and Body Composition Variables in Patients with Musculoskeletal Disorders: A Systematic Review and Meta-Analysis

**DOI:** 10.3390/jcm11236937

**Published:** 2022-11-24

**Authors:** José Casaña, Clovis Varangot-Reille, Joaquín Calatayud, Luis Suso-Martí, Enrique Sanchís-Sánchez, Ramón Aiguadé, Rubén López-Bueno, Pedro Gargallo, Ferran Cuenca-Martínez, María Blanco-Díaz

**Affiliations:** 1 Exercise Intervention for Health Research Group (EXINH-RG), Department of Physiotherapy, University of Valencia, 46010 Valencia, Spain; 2 Nursing and Physiotherapy Department, University of Lleida, 25008 St. Lleida, Spain; 3 Health Care Research Group (GRECS), Biomedical Research Institute of Lleida, 25198 St. Lleida, Spain; 4 Department of Physical Medicine and Nursing, University of Zaragoza, 50009 Zaragoza, Spain; 5 Department of Physiotherapy, Faculty of Medicine and Health Science, Catholic University of Valencia, 46001 Valencia, Spain; 6 Surgery and Medical Surgical Specialties Department, Faculty of Medicine and Health Sciences, University of Oviedo, 33003 Oviedo, Spain

**Keywords:** high-intensity interval training, musculoskeletal pain, body composition, blood pressure, heart rate

## Abstract

In order to assess the impact of high-intensity interval training (HIIT) on biological and body composition variables in patients with musculoskeletal disorders (MSKD), a systematic search on PubMed (Medline), CENTRAL, CINAHL, Web of Science, SPORTDiscus, and Scopus was conducted. Standardized mean differences (SMD) and 95% confidence intervals were calculated and pooled in a meta-analysis using the random-effects model. The effectiveness of HIIT on waist circumference, muscle mass, resting heart rate, resting systolic and diastolic blood pressure, C-reactive protein, body weight, and body fat were determined. GRADE, risk of bias 2, and PEDro scales were employed. HIIT compared to no intervention, minimal intervention, or usual care did not show significant results in its favor on any of the variables studied, except for the resting heart rate when compared with no intervention (SMD = −0.33; 95% CI: −0.63, −0.04; heterogeneity Q value: 0.14; *p* = 0.93; I^2^ = 0%). In addition, HIIT also does not seem to be more effective than moderate-intensity continuous training. Based on the results, it seems that HIIT has almost no significant effects on biological and body composition variables, except for resting heart rate, in patients with MSKD.

## 1. Introduction

Musculoskeletal disorders (MSKD) are clinical entities that affect the human locomotor system, having a major impact on the functionality, disability, and quality of life of the patients who suffer from them [1]. They often present impaired psychological health, increased risk of developing other chronic conditions, and higher levels of all-cause mortality [1]. Briggs et al. [2] reported that approximately 30% of the world’s population suffers from a persistent MSKD accompanied by pain. Disability associated with musculoskeletal conditions has been increasing and it is expected to continue in the coming decades [2]. Therefore, it seems that MSKD has a major impact on the impact of people’s lives, with all that this entails on a social, economic, and personal level. Although high-intensity interval training (HIIT) is not an entirely novel exercise model, its use in the rehabilitation of patients with MSKD has been emerging in recent years. Andreato [3] commented that HIIT is a form of training that alternates high-intensity exercises at 90% of the maximal oxygen consumption (VO2 max) (or ≥80% of the VO2 max for the clinical population) with recovery periods, repeating the exercise several times. Recovery periods are low intensity (between 40–60% of VO2 max).

There are some systematic reviews and meta-analyses that analyzed the effect of HIIT on several chronic conditions: HIIT and cardiorespiratory fitness in overweight and obese adults [4], HIIT and cardiometabolic risk factors in childhood obesity [5], HIIT and metabolic parameters in women with polycystic ovary syndrome [6], or HIIT and the prehabilitation of cancer patients [7] among others. Therefore, several articles on HIIT have mainly focused on patients with cardiovascular, cancer, or obesity diseases [8,9,10]. However, no published review has assessed the effects of HIIT on biological and body composition variables in patients with MSKD. Wu et al. [11] conducted a systematic review and meta-analysis of the effects of HIIT on biological and body composition variables in older adults. They found that HIIT intervention induces favorable adaptions in cardiorespiratory fitness, physical fitness, muscle power, cardiac contractile function, mitochondrial citrate synthase activity, and reduced blood triglyceride and glucose levels. This led us to think that it might be interesting to conduct a similar review study but applied to patients with MSKD. However, it is important to stress that the population of Wu et al. [11] is of a different age than this research work, as they performed it in older people.

Therefore, the main aim of the present study was to assess the effectiveness of HIIT on muscle mass, resting heart rate, resting systolic and diastolic blood pressure, C-reactive protein, body weight, body fat, muscle mass, and waist circumference variables in patients with MSKD.

## 2. Methods

This systematic review and meta-analysis were performed according to the Preferred Reporting Items for Systematic Reviews and Meta-analysis (PRISMA) 2020 statement actualized by Page et al. [12].

### 2.1. Inclusion Criteria

The selection criteria used in this systematic review and meta-analysis were based on methodological and clinical factors, such as the Population, Intervention, Control, Outcomes, and Study design (PICOS) described by Stone [13].

#### 2.1.1. Population

The participants selected for the studies were patients older than 18 years with any kind of MSKD. The participants’ gender was irrelevant.

#### 2.1.2. Intervention and Control

The intervention was the HIIT exercise modality, which could be given as an independent treatment, added to an existing intervention, or embedded in an existing intervention (e.g., usual treatment and care). For the control group, the comparators were minimal intervention, no intervention, and usual care (e.g., maintenance of the habitual daily activity profile, standard recommendations, or physical exercise habits) in combination or not with placebo interventions. In addition, a sub-analysis was performed to evaluate the effectiveness of HIIT compared with moderate-intensity continuous training (MICT) in those articles that, in addition to control or comparator with no intervention or minimal intervention, presented an additional group that performed this exercise model.

#### 2.1.3. Outcomes

The measures used to assess the results and effects were waist circumference, muscle mass, resting heart rate, resting systolic and diastolic blood pressure, C-reactive protein, body weight, and body fat.

#### 2.1.4. Study Design

Randomized controlled trials (RCTs), randomized parallel-design controlled trials, randomized cross-over trials, and prospective controlled clinical trials were selected. There was no restriction for any study design that had an intervention.

### 2.2. Search Strategy

The search for studies was performed using Medline (PubMed), Cochrane Central Register of Controlled Trials (CENTRAL), Cumulative Index to Nursing and Allied Health Literature (CINAHL), Web of Science, SPORTDiscus, and Scopus, from inception to the last search on 17 August 2021. A validated search filter for retrieving studies on measurement properties in PubMed was used; the same filter was adapted for all other databases [14]. The search was adapted and performed in Google Scholar due to its capacity to search for relevant articles and grey literature [15,16]. No restrictions were applied to any specific language as recommended by the international criteria [17]. The search strategy used in Medline (PubMed) combined medical subject headings (MeSH) and non-MeSH terms, adding a Boolean operator (OR and/or AND) to combine them. The search strategy was adapted to other electronic databases. The different search strategies used are detailed in Appendix A.

Two independent reviewers (CVR and FCM) conducted the search using the same methodology, and the differences were resolved by consensus moderated by a third reviewer (JCG). Additionally, meticulous manual searches were performed, including journals that have published articles related to the topic of this review as well as reference lists of the included studies. The reference sections of the original studies were screened manually [18].

### 2.3. Selection Criteria and Data Extraction

First, two independent reviewers (CVR and FCM), who assessed the relevance of the RCTs regarding the study questions and aims, performed a data analysis, which was performed based on information from the title, abstract, and keywords of each study. If there was no consensus or the abstracts did not contain sufficient information, the full text was reviewed. In the second phase of the analysis, the full text was used to assess whether the studies met all the inclusion criteria. Differences between the two independent reviewers were resolved by a consensus process moderated by a third reviewer (JCG) [19]. Data described in the results were extracted by means of a structured protocol that ensured that the most relevant information was obtained from each study [20].

### 2.4. Risk of Bias Assessment

The risk of bias 2 (RoB 2) tool and its adaption for cross-over trials was used to assess randomized trials [21]. It covers a total of five domains: (1) bias arising from the randomization process, (2) bias due to deviations from the intended interventions, (3) bias due to missing outcome data, (4) bias in measurement of the outcome, and (5) bias in selection of the reported result. The adaptation for cross-over trials has a supplementary adapted domain: (6) bias arising from period and carryover [22]. The study will be categorized as having (a) low risk of bias if all domains show low risk of bias, (b) some concerns if one domain is rated with some concerns without any with high risk of bias, and (c) high risk of bias if one domain is rated as having high risk of bias or multiple with some concerns.

Two independent reviewers (CVR and FCM) examined the quality of all the selected studies using the same methodology. Disagreements between the reviewers were resolved by consensus with a third reviewer (JCG). The concordance between the results (inter-rater reliability) was measured using Cohen’s kappa coefficient (κ) as follows: (1) κ > 0.7 indicated a high level of agreement between assessors; (2) κ = 0.5–0.7 indicated a moderate level of agreement; and (3) κ < 0.5 indicated a low level of agreement) [23].

### 2.5. Methodological Quality Assessment

The study’s methodological quality was assessed using the PEDro scale [24], which assesses the internal and external validity of a study and consists of 11 criteria: (1) specified study eligibility criteria, (2) random allocation of patients, (3) concealed allocation, (4) measure of similarity between groups at baseline, (5) patient blinding, (6) therapist blinding, (7) assessor blinding, (8) fewer than 15% dropouts, (9) intention-to-treat analysis, (10) intergroup statistical comparisons, and (11) point measures and variability data. The methodological criteria were scored as follows: yes (1 point), no (0 points), or do not know (0 points). The PEDro score for each selected study provided an indicator of the methodological quality (9–10 = excellent; 6–8 = good; 4–5 = fair; 3–0 = poor) [25]. The data obtained from the PEDro scale were used to map the results of the quantitative analyses.

Two independent reviewers (CVR and FCM) examined the quality of all the selected studies using the same methodology. Disagreements between the reviewers were resolved by consensus with a third reviewer (JCG). The concordance between the results (inter-rater reliability) was measured using Cohen’s kappa coefficient (κ) as follows: (1) κ > 0.7 indicated a high level of agreement between assessors; (2) κ = 0.5–0.7 indicated a moderate level of agreement; and (3) κ < 0.5 indicated a low level of agreement) [23].

### 2.6. Certainty of Evidence

The certainty of evidence analysis was based on classifying the results into levels of evidence according to the Grading of Recommendations, Assessment, Development and Evaluation (GRADE) framework, which is based on five domains: study design, imprecision, indirectness, inconsistency, and publication bias [26]. The assessment of the five domains was conducted according to GRADE criteria [27,28]. Evidence was categorized into the following four levels accordingly: (a) *High quality.* Further research is very unlikely to change our confidence in the effect estimate. All five domains are also met: (b) *Moderate quality.* Further research is likely to have an important impact on our confidence in the effect estimate and might change the effect estimate. One of the five domains is not met: (c) *Low quality.* Further research is very likely to have a significant impact on our confidence in the effect estimate and is likely to change the estimate. Two of the five domains are not met: (d) *Very low quality.* Any effect estimates highly uncertain. Three of the five domains are not met [27,28].

For the study design domain, the recommendations were downgraded one level in the event there was an uncertain or high risk of bias and serious limitations in the effect estimate (more than 25% of the participants were from studies with fair or poor methodological quality, as measured by the PEDro scale). In terms of inconsistency, the recommendations were downgraded one level when the point estimates varied widely among studies, the confidence intervals showed minimal overlap, or when the I^2^ was substantial or large (greater than 50%). At indirectness, domain recommendations were downgraded when severe differences in interventions, study populations, or outcomes were found (the recommendations were downgraded in the absence of direct comparisons between the interventions of interest or when there are no key outcomes, and the recommendation is based only on intermediate outcomes or if more than 50% of the participants were outside the target group). For the imprecision domain, the recommendations were downgraded one level if there were fewer than 300 participants for the continuous data [29]. Finally, recommendations were downgraded due to the strong suspicion of publication bias by Doi plot and LFK index (i.e., LFK index > 2 or LFK index < −2).

### 2.7. Data Synthesis and Analysis

The statistical analysis was conducted using *MetaXL* software (version 5.3, EpiGear International, Sunrise Beach, QLD, Australia) [30]. To compare the outcomes reported by the studies, the standardized mean difference (SMD) was calculated over time, as well as the corresponding 95% confidence interval (CI) for the continuous variables. The statistical significance of the pooled SMD was examined as Hedges’ g to account for a possible overestimation of the true population effect size in the small studies [31].

When data were expressed as a median and interquartile range, they were transformed into mean and standard deviation (SD) according to Wan’s method [32]. When data were expressed as within-group mean difference and CI, CI was transformed into SD according to the formula recommended by the Cochrane Handbook for Systematic Reviews of Interventions version 6.2: SD = √(N) × (upper limit − lower limit)/3.92 [33].

The same inclusion criteria were used for the systematic review and the meta-analysis and included three additional criteria: (1) in the results, they were detailed information regarding the comparative statistical data of the exposure factors, therapeutic interventions, and treatment responses; (2) the intervention was compared with a similar control group; and (3) data on the analyzed variables were represented in at least three studies.

The estimated SMDs were interpreted as described by Hopkins et al. [34], considering that an SMD of 4.0 represented an extremely large clinical effect, 2.0–4.0 represented a very large effect, 1.2–2.0 represented a large effect, 0.6–1.2 represented a moderate effect, 0.2–0.6 represented a small effect, and 0.0–0.2 represented a trivial effect. The degree of heterogeneity among the studies was estimated using Cochran’s Q statistic test (a *p*-value < 0.05 was considered significant) and the inconsistency index (I^2^) [34]. It has been considered that an I^2^ > 25% represented small heterogeneity, I^2^ > 50% represented medium heterogeneity, and I^2^ > 75% represented large heterogeneity [35]. The I^2^ index is a complement to the Q test, although it has the same problems of power with a small number of studies [35]. When the Q test was significant (*p* < 0.1) and/or the result of I^2^ was >75%, there was heterogeneity among the studies, and the random-effects model was conducted in the meta-analysis. To detect publication bias and to test the influence of each individual study, a visual evaluation of the Doi plot [36] was performed, seeking asymmetry. In addition, a quantitative measure of the Luis Furuya-Kanamori (LFK) index was also performed, which has been shown to be more sensitive than the Egger test in detecting publication bias in a meta-analysis of a low number of studies [37]. An LFK index within ±1 represents no asymmetry, exceeding ±1 but within ±2 represents minor asymmetry, and exceeding ±2 involves major asymmetry.

## 3. Results

### 3.1. Characteristics of the Included Studies

The study strategy is shown in the form of a flow chart (Appendix A). From 429 studies initially detected, a total of eight studies were included, six randomized controlled trials [38,39,40,41,42,43,44] and one randomized cross-over trial [45]. Five were from Europe, [41,42,43,44,45] two from Oceania [39,40] and one from the Middle East [38]. A total of 380 participants with a mean age ranging from 30.2 to 62.4 years were included. The patients were mostly women (50–100%) diagnosed with fibromyalgia [38], persistent pain condition [39], knee osteoarthritis [40], rheumatoid arthritis [45], adults with juvenile idiopathic arthritis [45], axial spondyloarthritis [41,42], or psoriatic arthritis [43,44]. Details of the participant’s characteristics and studies are shown in Appendix A.

The studies compared HIIT training against no intervention, usual care, yoga, or moderate-intensity continuous training. The intervention duration ranged between 8 and 12 weeks. The frequency of training ranged mainly between two and three times per week, however, Keogh et al., and Atan and Karavelioğlu applied four times and five times, respectively [38,40]. Most of the studies used a HIIT protocol of four sets of 4-min intervals with 3 min of resting, for a work/rest ratio of 1:0.75 [38,41,42,43,45]. When reported, the intensity used in the HIIT protocol ranged between 85 and 95% of HR_max_ for the intervals and 70% of HR_max_ for the rest. Intervention characteristics of the studies included are presented in detail in Appendix A.

### 3.2. Methodological Quality and Risk of Bias Results

The methodological quality of the studies was evaluated with the PEDro scale. Regarding the methodological quality, they were all rated as having good methodological quality. The items worst scored were the blinding of patients and therapists. The PEDro scores for each study are shown in Appendix A. The inter-rater reliability of the methodological quality assessment between assessors was high (k = 0.88).

The risk of bias in randomized trials was evaluated with the RoB 2 tool and adaptation of the RoB 2 tool for cross-over trials. The domain with the highest percentage of studies with a high risk of bias is a deviation from the intended interventions (60%). The risk of bias summary of the randomized trial is shown in Appendix A. The inter-rater reliability of the risk of bias assessment between assessors was high (k = 0.845).

### 3.3. Qualitative Analysis

#### 3.3.1. HIIT Training against No Intervention, Minimal Intervention, or Usual Care

Two studies assessed the effect of HIIT training—against usual care or no intervention—on waist circumference [41,42,45]. Sandstad et al. found only statistically significant differences over time in the HIIT group [45]. Waist circumference results were not pooled because two studies only analyzed patients with baseline-increased circumference (males ≥94 cm and females ≥80 cm) [41,42]. In those patients, they found contrary results on the efficacy of HIIT training; nonetheless, Sveaas et al. included a larger sample in 2019 and found a significant treatment effect [41,42].

#### 3.3.2. HIIT Training against Moderate-Intensity Continuous Training

Two studies found that HIIT or MICT training do not seem to increase muscle mass in patients with MSKD [38,40]. MICT training is effective to decrease body fat and body weight in patients with fibromyalgia but not HIIT training [38], however, there was no difference in patients with knee osteoarthritis [40]. Atan and Karavelioğlu also found the use of HIIT and MICT training could decrease the resting heart rate and also systolic and diastolic blood pressure but without significant differences between groups [38].

### 3.4. Meta-Analysis Results

The overall quality of evidence is detailed in Appendix A.

#### HIIT Training against No Intervention, Minimal Intervention, or Usual Care

Resting Heart Rate Variable

**Figure 1:**
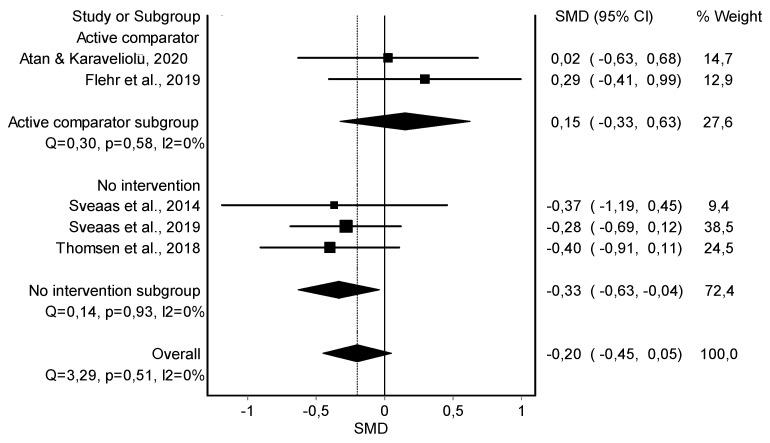
Synthesis forest plot of resting heart rate variable. If the diagram (diamond-shaped) is in the negative zone (on the left of the figure and without touching the 0 line), it indicates statistically significant differences in favor of the HIIT intervention. The forest plot summarizes the results of included studies (standardized mean differences [SMDs], and weight). The small boxes with squares represent the point estimate of the effect size and sample size. The lines on either side of the box represent a 95% confidence interval (CI).

A comparison was made by subgroups and one overall. Starting with the first analysis, the meta-analysis showed no significant differences in favor of HIIT when compared to an active comparator (yoga [39] or recommendations about exercise [38]) (*n* = 87; SMD = 0.15; 95% CI: −0.33, 0.63; heterogeneity Q value: 0.3; *p* = 0.58; I^2^ = 0%). However, the meta-analysis showed significant changes in favor of HIIT when compared against no intervention (*n* = 161; SMD = −0.33; 95% CI: −0.63, −0.04; heterogeneity Q value: 0.14; *p* = 0.93; I^2^ = 0%). Finally, overall, the meta-analysis showed no statistically significant differences in the resting heart rate in five studies [38,39,41,42,44] (*n* = 248; SMD = −0.20; 95% CI: −0.45, 0.05; heterogeneity Q value: 3.29; *p* = 0.51; I^2^ = 0%) showing no heterogeneity (Figure 1). The visual evaluation of the funnel and Doi plot showed a minor asymmetry (LFK index = 1.67) (Appendix A). A subgroup analysis showed statistically significant differences when HIIT training is compared with no intervention.

Resting Systolic Blood Pressure Variable

**Figure 2:**
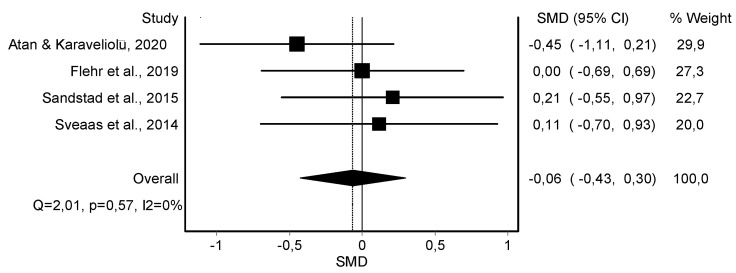
Synthesis forest plot of resting systolic blood pressure variable. If the diagram (diamond-shaped) is in the negative zone (on the left of the figure and without touching the 0 line), it indicates statistically significant differences in favor of the HIIT intervention. The forest plot summarizes the results of included studies (SMD and weight). The small boxes with squares represent the point estimate of the effect size and sample size. The lines on either side of the box represent a 95% CI.

The meta-analysis showed no statistically significant differences in the resting systolic blood pressure in four studies [38,39,42,45] (*n* = 119; SMD = −0.06; 95% CI: −0.43, 0.30; heterogeneity Q value: 2.01; *p* = 0.57; I^2^ = 0%) showing no heterogeneity (Figure 2). The visual evaluation of the funnel and Doi plot showed no asymmetry (LFK index = 0.00) (Appendix A).

Resting Diastolic Blood Pressure Variable

**Figure 3:**
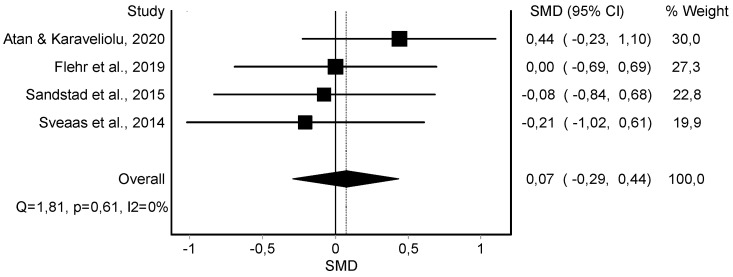
Synthesis forest plot of resting diastolic blood pressure variable. If the diagram (diamond-shaped) is in the negative zone (on the left of the figure and without touching the 0 line), it indicates statistically significant differences in favor of the HIIT intervention. The forest plot summarizes the results of included studies (SMD and weight). The small boxes with squares represent the point estimate of the effect size and sample size. The lines on either side of the box represent a 95% CI.

The meta-analysis showed no statistically significant differences in the resting diastolic blood pressure in four studies [38,39,42,45] (*n* = 119; SMD = 0.07; 95% CI: −0.29, 0.44; heterogeneity Q value: 1.81; *p* = 0.61; I^2^ = 0%) showing no heterogeneity (Figure 3). The visual evaluation of the funnel and Doi plot showed no asymmetry (LFK index = −0.02) (Appendix A).

Body Weight Variable

The meta-analysis showed no statistically significant differences in body weight in 4 studies [38,41,42,45] (*n* = 183; SMD = −0.34; 95% CI: −0.80, 0.12; heterogeneity Q value: 6.12; *p* = 0.11; I^2^ = 51%) showing no heterogeneity (Appendix A). The visual evaluation of the funnel and Doi plot showed major asymmetry (LFK index = −2.25) (Appendix A).

Body Fat Variable

The meta-analysis showed no statistically significant differences in body fat in four studies [38,42,44,45] (*n* = 148; SMD = −0.24; 95% CI: −0.57, 0.08; heterogeneity Q value: 2.47; *p* = 0.48; I^2^ = 0%) showing no heterogeneity (Appendix A). The visual evaluation of the funnel and Doi plot showed major asymmetry (LFK index = 3.41) (Appendix A).

Muscle Mass Variable

The meta-analysis showed no statistically significant differences in body fat in three studies [38,44,45] (*n* = 124; SMD = 0.04; 95% CI: −0.32, 0.39; heterogeneity Q value: 0.01; *p* = 0.99; I^2^ = 0%) showing no heterogeneity (Appendix A). The visual evaluation of the funnel and Doi plot showed minor asymmetry (LFK index = −1.11) (Appendix A).

C-reactive Protein Variable

**Figure 4:**
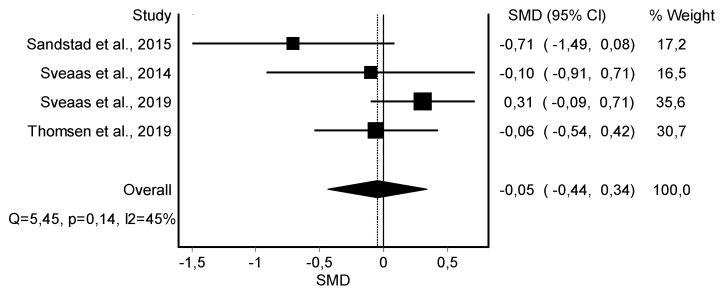
Synthesis forest plot of C-reactive protein variable. If the diagram (diamond-shaped) is in the negative zone (on the left of the figure and without touching the 0 line), it indicates statistically significant differences in favor of the HIIT intervention. The forest plot summarizes the results of included studies (SMD and weight). The small boxes with squares represent the point estimate of the effect size and sample size. The lines on either side of the box represent a 95% CI.

The meta-analysis showed no statistically significant differences in the C-reactive protein in four studies [41,42,43,45] (*n* = 215; SMD = −0.11; 95% CI: −0.44, 0.34; heterogeneity Q value: 5.45; *p* = 0.14; I^2^ = 45%) showing no heterogeneity (Figure 4). The visual evaluation of the funnel and Doi plot showed minor asymmetry (LFK index = −1.75) (Appendix A).

## 4. Discussion

The main aim of the present study was to assess the impact of HIIT on biological and body composition variables in patients with MSKD. The main results showed that HIIT intervention compared to no intervention, minimal intervention, or usual care did not show significant results in its favor on any of the variables studied, except for the resting heart rate when compared with no intervention. In addition, the HIIT intervention also did not show significant results when compared with MICT.

In recent years, a significant body of evidence on HIIT has developed. Recently, it was found that HIIT can improve insulin sensitivity, blood pressure, and body composition in adults, with a moderate level of evidence [46]. Benefits have also been shown with HIIT in patients with neurological pathologies [47] or in cardiac rehabilitation [48]. However, no significant differences were found between HIIT and continuous MICT [46]. The results obtained in the present study are in line with the current literature regarding the resting heart rate variables, showing benefits for HIIT intervention compared to no intervention but showing similar results to MICT. In this sense, considering that previous reviews estimate that HIIT involved ~40% less time commitment than MICT and also demonstrated a comparable dropout rate, it has been suggested that HIIT may be a time-efficient and sustainable strategy to induce improvements in several interesting variables such pain intensity or VO2 max [49,50]. Epidemiological data have suggested a significant association between higher morbidity and mortality and increased resting heart rate [51]. In addition, it appears that the heart functions more efficiently by needing fewer beats per minute to oxygenate all parts of the body when the resting heart rate is reduced [52].

One of the most relevant findings found in the present study is the absence of statistically significant changes in variables related to metabolic health, such as body weight or fat percentage. In this regard, previous research has shown that the prevalence of being overweight is high in patients with musculoskeletal pain, and this may be associated with pain intensity, disability, and/or quality of life [53]. For this reason, physical exercise is strongly recommended, which could help to maintain healthy body composition values and reduce musculoskeletal pain [54]. In a previous systematic review and meta-analysis, HIIT and MICT showed similar efficacy in all body composition measurements (such as whole-body fat mass, fat loss, or waist circumference) in patients with obesity, but HIIT may be a time-efficient component of weight management programs [49]. They found moderate clinical evidence in favor of both exercise models [49]. However, in our study, these changes were not found in patients with musculoskeletal pain. Several reasons could explain this finding. First of all, previous studies questioned the existence of non-responders to HIIT and emphasized the need to train at adequate training intensity [55]. This may be especially relevant in patients with musculoskeletal pain, who could have difficulty following high-intensity exercise, reducing adherence and training effects [56,57]. In addition, the studies which randomly allocated participants to an exercise model reported no significant change in body composition. However, one study that allowed participants to choose their exercise reported significant reductions in body mass and waist circumference [58,59]. Future studies should consider variables such as adherence or the preferences of the patient with musculoskeletal pain when prescribing exercise. In addition, results may be explained by sources of variability between individuals, including behavioral or environmental changes and nutritional status, aspects that should be considered in future research.

The present paper has a number of limitations that must be taken into consideration. First, further studies are needed on the effects of HIIT on MSKD to confirm our results. The sample sizes of the included studies were often very small. Future studies should include larger sample sizes to improve the quality of the evidence. Due to the lack of sufficient data and the heterogeneity among the interventions (e.g., frequency, intervention duration), we could not establish the specific effect on each MSKD and the optimal HIIT parameters. Readers should be aware that it is likely that patients with MSKD could not reach the required intensity in the included studies and thus could not achieve the expected adaptations. For instance, some studies adapted the protocol when the pain reached a certain intensity, which presumably would reduce cardiorespiratory stimulus, whereas other studies did not control pain intensity during HIIT. It is important to stress that there were studies where HIIT was embedded in other exercise interventions such as strength training, balance, or continuous exercise. Future studies aimed to compare the effectiveness of HIIT with other exercise types should evaluate them separately. Furthermore, some studies did not clearly report the whole exercise protocol, for example without mentioning exercise intensity. These are clear limitations that should be considered when extrapolating the results. Future studies should try to standardize nomenclature and data reports. In spite of the aforementioned limitations, the present study provides novel evidence for the use of HIIT in patients with MSKD.

## 5. Conclusions

In conclusion, results showed that HIIT has no statistically significant impact on waist circumference, muscle mass, resting systolic and diastolic blood pressure, C-reactive protein, body weight, and body fat, except resting heart rate, in patients with MSKD. It is important to take the results obtained with caution due to the small number of trials, the heterogeneity of the HIIT workouts analyzed, as well as the large number of reported limitations.

## Figures and Tables

**Figure A1:**
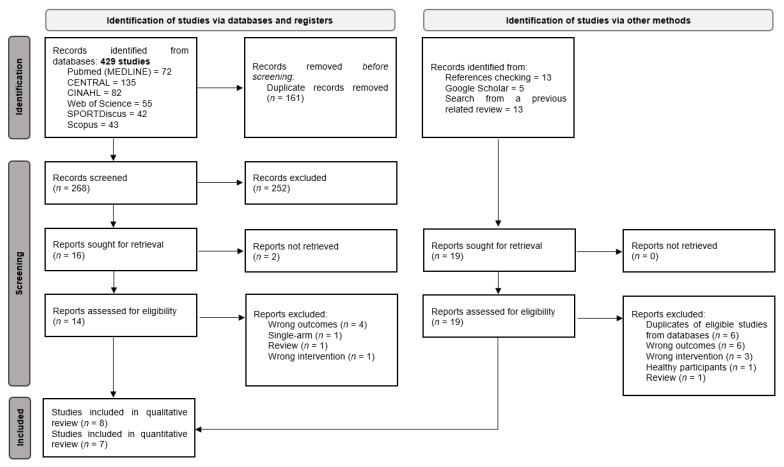
PRISMA 2020 flowchart of studies selection.

**Figure A2:**
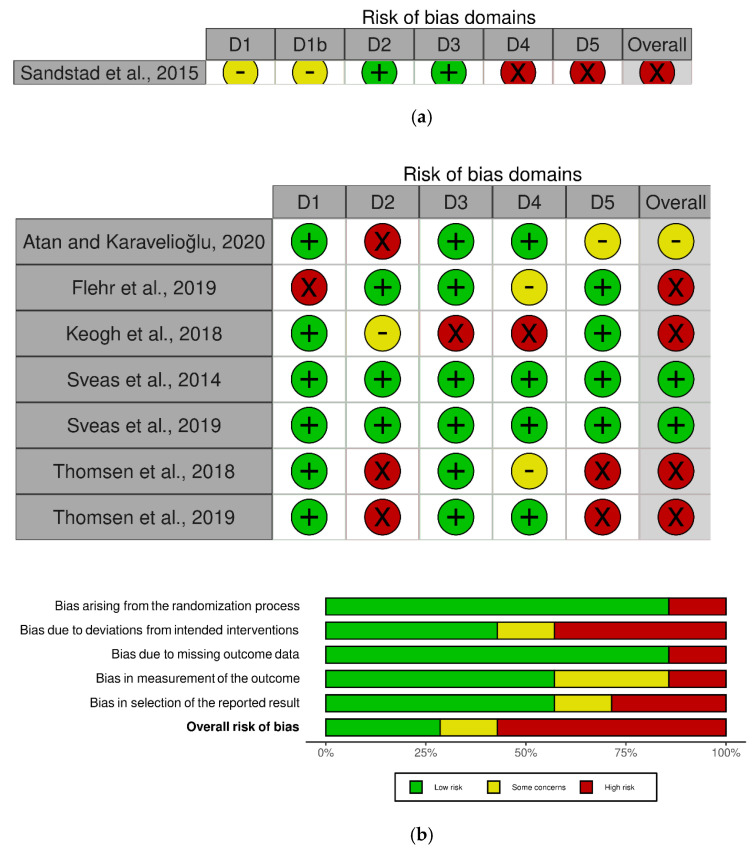
Risk of bias summary. Review authors’ judgements about each risk of bias item for each included study (risk of bias 2 tool) and risk of bias graph. Review authors’ judgements about each risk of bias item presented as percentages across all included studies (risk of bias 2 tool). (**a**) D1: randomization process, D2: deviations from the intended interventions, D3: missing outcome data, D4: measurement of the outcome D5: selection of the reported result; (**b**) D1: randomization process, D2: deviations from the intended interventions, D3: missing outcome data, D4: measurement of the outcome, D5: selection of the reported result.

**Figure A3:**
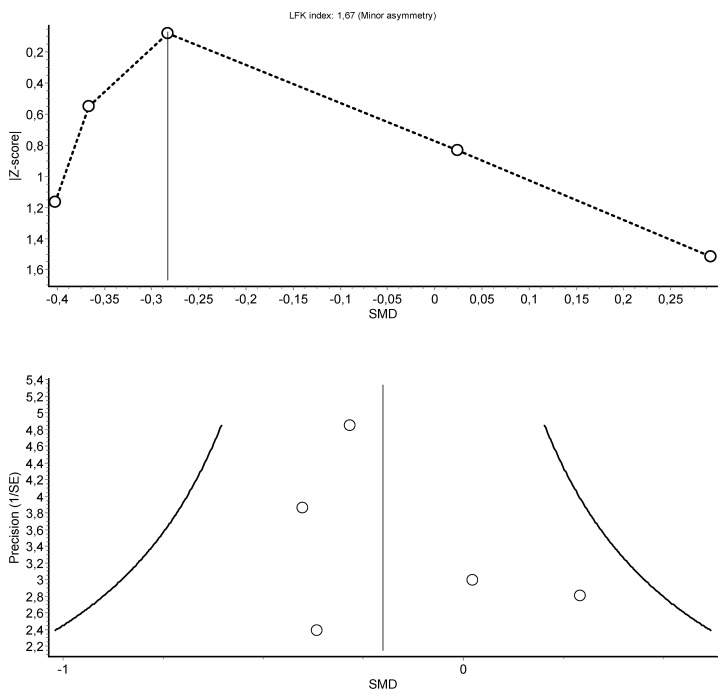
Synthesis funnel and Doi plot (LFK index) for resting heart rate.

**Figure A4:**
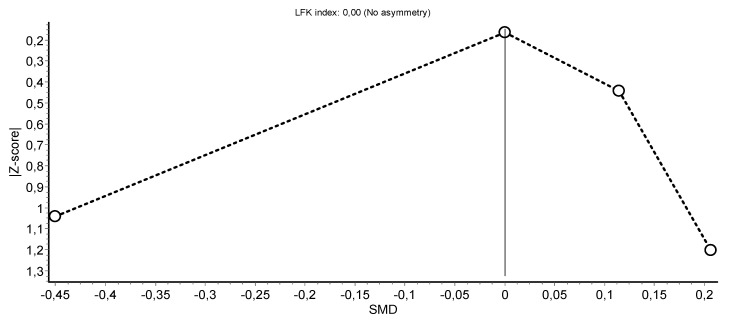

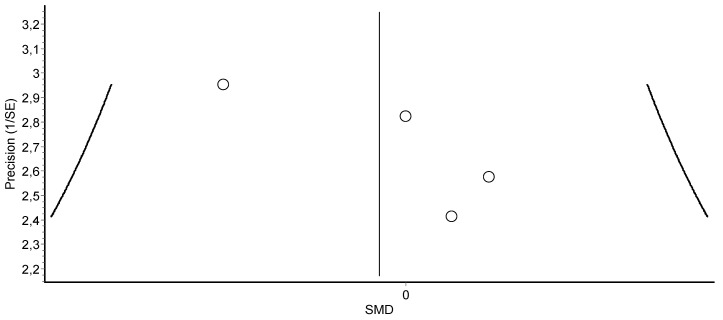
Synthesis funnel and Doi plot (LFK index) for resting systolic blood pressure.

**Figure A5:**
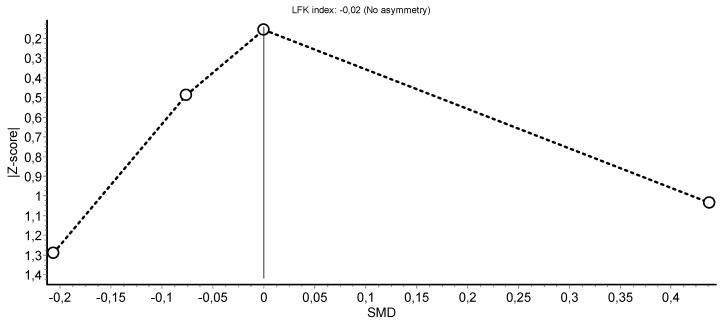

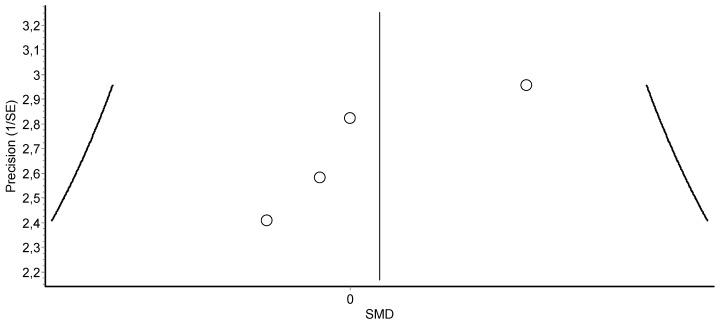
Synthesis funnel and Doi plot (LFK index) for resting diastolic blood pressure.

**Figure A6:**
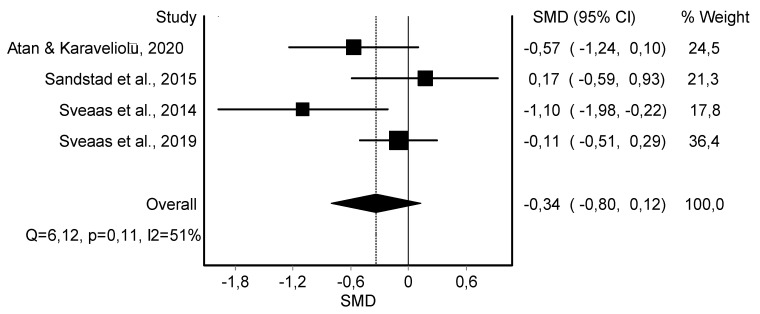
Synthesis forest plot of body weight. Negative favor HIIT intervention. The forest plot summarizes the results of included studies (standardized mean differences [SMDs] and weight). The small boxes with the squares represent the point estimate of the effect size and sample size. The lines on either side of the box represent a 95% confidence interval (CI).

**Figure A7:**
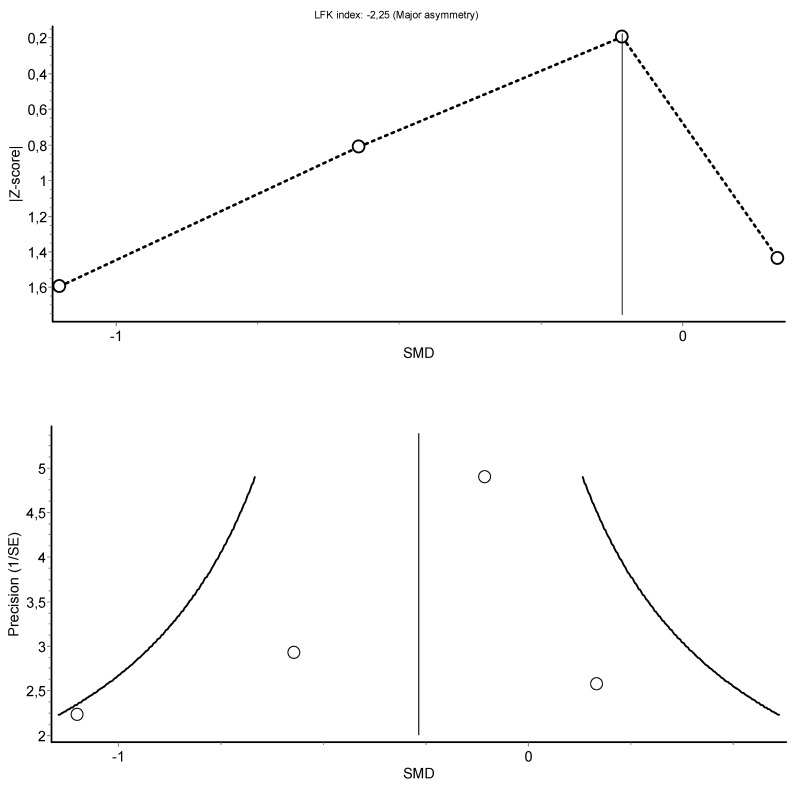
Synthesis funnel and Doi plot (LFK index) for body weight.

**Figure A8:**
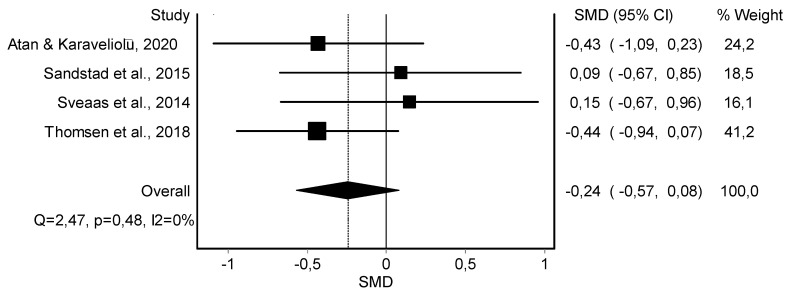
Synthesis forest plot of body fat variable. Negative favor HIIT intervention. The forest plot summarizes the results of included studies (standardized mean differences [SMDs] and weight). The small boxes with the squares represent the point estimate of the effect size and sample size. The lines on either side of the box represent a 95% confidence interval (CI).

**Figure A9:**
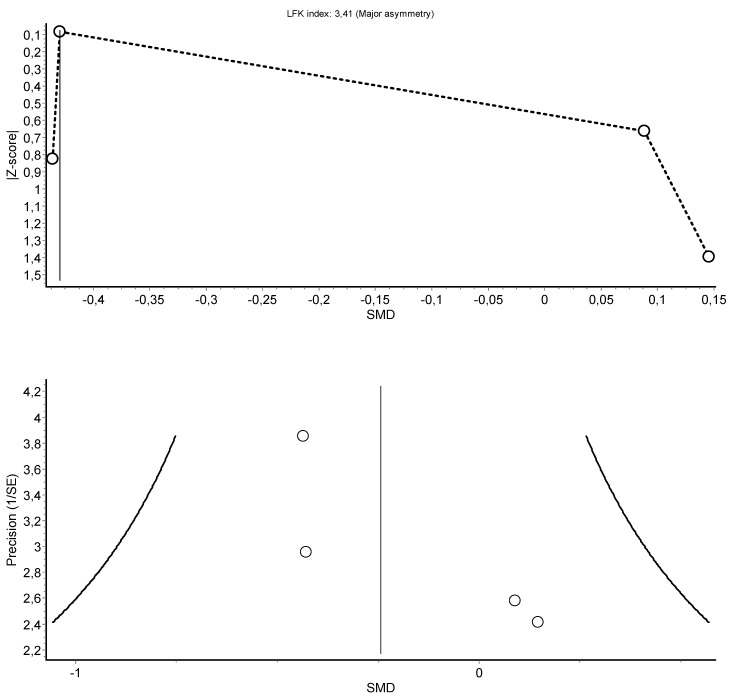
Synthesis funnel and Doi plot (LFK index) for body fat.

**Figure A10:**
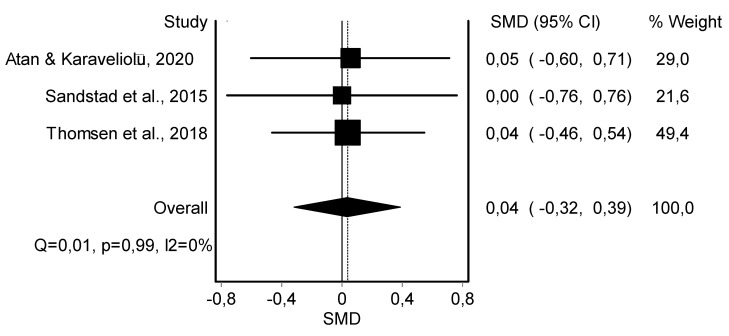
Synthesis forest plot of muscle mass variable. Positive favor HIIT intervention. The forest plot summarizes the results of included studies (standardized mean differences [SMDs] and weight). The small boxes with the squares represent the point estimate of the effect size and sample size. The lines on either side of the box represent a 95% confidence interval (CI).

**Figure A11:**
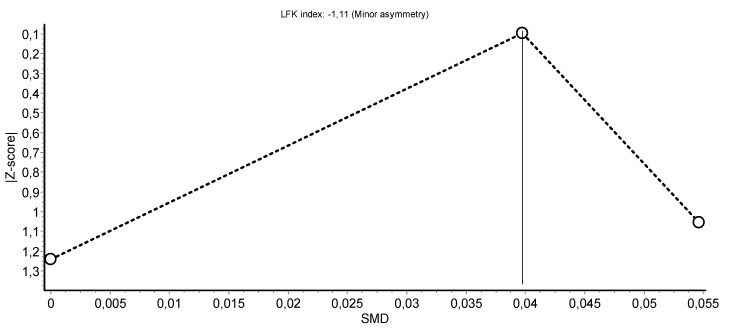

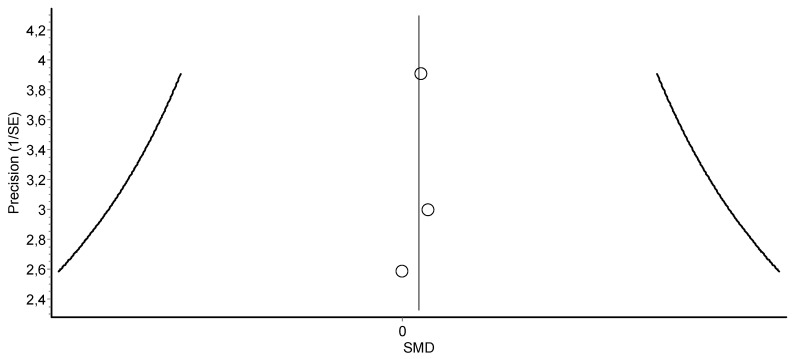
Synthesis funnel and Doi plot (LFK index) for muscle mass.

**Figure A12:**
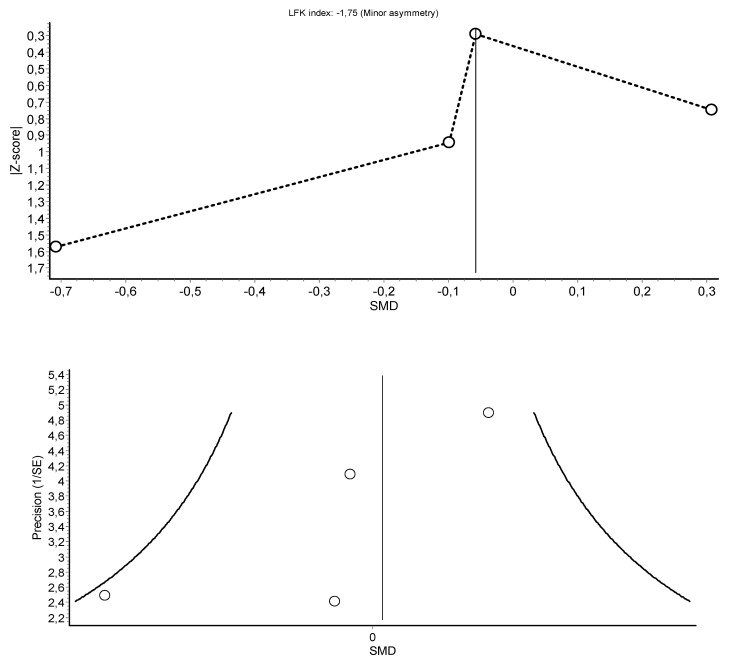
Synthesis funnel and Doi plot (LFK index) for C-reactive protein.

## Data Availability

Data is available on request from the corresponding author.

## Appendix A. Search Strategy in the Different Electronic Databases

**PubMed (MEDLINE)—72 trials**

((“high intensity interval training”) OR (“high-intensity interval training”) OR (“HIIT”) OR (“H.I.I.T.“) OR (“High-Intensity Interval Training”[Mesh])) AND ((“pain”) OR (“musculoskeletal disorder”) OR (“musculoskeletal disorders”) OR (“chronic pain”) OR (“Musculoskeletal Pain”[Mesh]) OR (“Chronic Pain”[Mesh])) AND ((“inflammatory marker”) OR (“pain”) OR (“inflammatory markers”) OR (“c-reactive protein”) OR (“body composition”) OR (“body weight”) OR (“body fat”) OR (“muscle mass”) OR (“heart rate”) OR (“blood pressure”) OR (“C-Reactive Protein”[Mesh]) OR (“Body Composition”[Mesh]) OR (“Heart Rate”[Mesh]) OR (“Blood Pressure”[Mesh])).

**Cochrane** **Central Register of Controlled Trials** (**CENTRAL**) **—135 trials**

**ID**	**Search Strategy**
**#1**	MeSH descriptor: [High-Intensity Interval Training] explode all trees
**#2**	(high intensity interval training) OR (HIIT) OR (High-intensity interval training)
**#3**	#1 OR #2
**#4**	MeSH descriptor: [Musculoskeletal Pain] explode all trees
**#5**	MeSH descriptor: [Chronic Pain] explode all trees
**#6**	(Pain) OR (musculoskeletal pain) OR (musculoskeletal disorder) OR (chronic pain)
**#7**	#4 OR #5 OR #6
**#8**	MeSH descriptor: [C-Reactive Protein] explode all trees
**#9**	MeSH descriptor: [Body Composition] explode all trees
**#10**	MeSH descriptor: [Heart Rate] explode all trees
**#11**	MeSH descriptor: [Blood Pressure] explode all trees
**#12**	(inflammatory marker) OR (pain) OR (inflammatory markers) OR (c-reactive protein) OR (body composition) OR (body weight) OR (body fat) OR (muscle mass) OR (heart rate) OR (blood pressure)
**#13**	#8 OR #9 OR #10 OR #11 OR #12
**#14**	#3 AND #7 AND #13

**Web of Science—82 trials**

TS = (high intensity interval training OR hiit OR high-intensity interval training) AND TS = (musculoskeletal disorder* OR musculoskeletal pain OR chronic pain OR pain) AND TS = (body composition* OR body fat OR muscle mass OR body weight OR inflammatory marker* OR c-reactive protein OR heart rate OR blood pressure).

**Cumulative Index to Nursing and Allied Health Literature (CINAHL)—55 trials**

(high intensity interval training or hiit or high intensity exercise or high intensity workout) AND (musculoskeletal disorders or musculoskeletal pain or musculoskeletal injuries or chronic pain or pain) AND (inflammatory markers or c-reactive protein or body composition or body fat or muscle mass or body weight or heart rate or blood pressure).

**SPORTDiscus—42 trials**

(high intensity interval training or hiit or high intensity exercise or high intensity workout) AND (musculoskeletal disorders or musculoskeletal pain or musculoskeletal injuries or chronic pain or pain) AND (inflammatory markers or c-reactive protein or body composition or body fat or muscle mass or body weight or heart rate or blood pressure).

**Scopus—43 trials**

(TITLE-ABS-KEY ( (“high intensity interval training” ) OR {high intensity interval training} OR ( “hit” ) ) AND TITLE-ABS-KEY ( ( “musculoskeletal pain” ) OR ( “musculoskeletal disorder*” ) OR ( “chronic pain” ) OR ( “pain” ) ) AND TITLE-ABS-KEY ( ( “inflammatory marker*” ) OR ( “c-reactive protein” ) OR ( “body composition” ) OR ( “body weight” ) OR ( “body fat” ) OR ( “muscle mass” ) OR ( “heart rate” ) OR ( “blood pressure” ) ) ).

**Google Scholar**

(“high intensity interval training” OR HIIT) AND (“musculoskeletal disorder*” OR “musculoskeletal pain” OR “chronic pain” or “pain”) AND (“inflammatory marker*” OR “c-reactive protein” OR “body composition” OR “heart rate” OR “blood pressure”).

**Table A1:** Characteristics of the included studies.

Author, Year Study Design Country	Population Disease (*n*) Age (Years) Gender (%) Diagnostic Criteria Disease Duration (Years)	Duration Intervention(s) and Control Group (*n*)	Outcome Measured (Units)	Results
**Atan and Karavelioğlu, 2020** [38] **Pilot RCT** Turkey	**Fibromyalgia** (*n* = 55) *Age:* 48.7 ± 9.1 yrs 100% F American College of Rheumatology 2016 diagnostic criteria *Duration:* 2.5 ± 1.6 yrs	6 weeks *Intervention* -HIIT (*n* = 19) -MICT (*n* = 19) *Control* Usual care (*n* = 17)	Resting SBP (mmHg) Resting DBP (mmHg) Resting HR (bpm) Body Fat % Body Weight (kg) Muscle Mass (kg)	Statistically significant decrease in the MICT group of body weight (*p* = 0.006), resting SBP (*p* = 0.018), resting HR (*p* = 0.018), and BMI (*p* = 0.008). Statistically significant decrease in the HIIT group of resting SBP (*p* = 0.049) and resting HR (*p* = 0.024).
**Flehr et al., 2019** [39] **RCT** Australia	**Persistent pain condition** (*n* = 32) *Age:* 30.2 ± 8 yrs 100% F N/R *Duration:* more than 12 months	8 weeks *Intervention* HIIT (*n* = 15) *Control* Bikram Yoga (*n* = 17)	Resting SBP (mmHg) Resting HBP (mmHg) Resting HR (bpm)	No statistically significant differences in any variables.
**Keogh et al., 2018** [40] **Pilot RCT** Australia	**Knee OA** (*n* = 17) *Age:* 62.4 ± 8.3 yrs 76% F/24% M Diagnostic by an orthopaedic surgeon *Duration*: 4.7 ± 4.6 yrs	8 weeks *Intervention* HIIT (*n* = 9) *Control* MICT (*n* = 8)	Body Fat % Body Weight (kg) Muscle Mass (kg)	No statistically significant differences in any variables.
**Sandstad et al., 2015** [45] **rCOT** Norway	**RA and JIA** (*n* = 27) *Age:* 33.0 ± 8.1 yrs 100% F Diagnosis by a rheumatologist *Duration:* N/R	10 weeks *Intervention* HIIT (*n* = 12) *Control* No intervention (*n* = 15)	Resting SBP (mmHg) Resting DBP (mmHg) Body Fat % Body Weight (kg) BMI (kg/m^2^) Waist Circumference (cm) Muscle Mass (%) hsCRP (mg/L)	Statistically significant differences in the HIIT group in BMI (*p* = 0.04), body fat (*p* = 0.04), muscle mass (*p* = 0.03), and waist circumference (*p* = 0.004). There was a trend toward decrease in hsCRP (*p* = 0.08). No statistically significant differences in blood pressure.
**Sveaas et al., 2014** [42] **Pilot RCT** Norway	**axSpA** (*n* = 24) *Age:* 48.5 ± 12.0 yrs 50% F/50% M Spondyloarthritis international society criteria *Duration:* 24.9 ± 15.8 yrs	12 weeks *Intervention* HIIT (*n* = 10) *Control* Usual care (*n* = 14)	Resting SBP (mmHg) Resting DBP (mmHg) Resting HR (bpm) Body Fat % Body Weight (kg) Waist Circumference (cm) CRP (mg/L)	Statistically significant differences in resting HR (*p* = 0.02), waist circumference (*p* = 0.02), and body fat (*p* < 0.001). No statistically significant differences in body weight, BMI, CRP, and blood pressure.
**Sveaas et al., 2019** [41] **RCT** **Norway**	**axSpA** (*n* = 97) *Age*: 46.2 ± N/R yrs 53% F/47% M Spondyloarthritis international society criteria Duration: N/R	12 weeks *Intervention* HIIT (*n* = 48) *Control* No intervention (*n* = 49)	Resting HR (bpm) Body Weight (kg) BMI (kg/m^2^) Waist Circumference (cm) CRP (mg/L)	Statistically significant decrease in CRP (*p* = 0.041). No statistically significant differences in resting HR, body weight, BMI, and waist circumference.
**Thomsen et al., 2018** [44] **RCT** Norway	**PsA** (*n* = 61) *Age:* 47.7 ± 11.9 yrs 67% F/33% M Classification of psoriatic arthritis study group criteria *Duration:* 6.2 ± 7.4 yrs	11 weeks *Intervention* HIIT (*n* = 30) *Control* No intervention (*n* = 31)	Body Fat (%) Resting HR (bpm) Lean Muscle Mass (g)	Participants in the HIIT group had a statistically significant decrease in their resting heart rate (*p* = 0.004) and body fat (*p* = 0.001), however, there were no statistically significant differences with control group.
**Thomsen et al., 2019** [43] **RCT** Norway	**PsA** (*n* = 67) *Age:* 48.0 ± 11.5 yrs 64% F/36% M Classification of psoriatic arthritis study group criteria *Duration:* N/R	11 weeks *Intervention* HIIT (*n* = 32) *Control* No intervention (*n* = 35)	hsCRP (mg/L)	No statistically significant differences in hsCRP.

Abbreviations: axSpA, axial spondyloarthritis; BMI, body mass index; CRP, C-reactive protein; DBP, diastolic blood pressure; HIIT, high-intensity interval training; HR, heart rate; hsCRP, highly sensitive C-reactive protein; JIA, juvenile idiopathic arthritis; MICT, moderate-intensity continuous training; N/R, not reported; OA, osteoarthritis; PsA, psoriatic arthritis; RA, rheumatoid arthritis; RCT, randomized control trial; rCOT, randomized cross-over trial; SBP, systolic blood pressure.

**Table A2:** Prescription parameters extracted from each included studies.

Trial	Group	Exercise Protocol (Distribution and Exercise Type)	Intensity (Pain Control during Training)	Frequency and Duration	Exercise Testing
**Atan and Karavelioğlu, 2020** [38]	HIIT + StrT + Stretching	*Total Exercise Duration:* 35 min *Warm-up and Cool-Down:* 5-min stationary cycling. *HIIT Protocol:* 4 × 4 min of high-intensity stationary cycling alternating with 3-min cycling recovery periods. *Work/Rest Ratio*: [1:0.75] Followed by 10-min full body (shoulder, arm, leg, and hip) StrT, using 1–3 kg weights (1 × 8–10 rep), and 5-min stretching (4–5 × 20–30 s for each muscle group).	*Measurement:*HR_max_ (Monitorization: N/R) *Warm-Up and Cool-Down:* 50% HR_max_ *HIIT:* *Interval:* 80–95% HR_max_ *Active Rest:* 70% HR_max_ *StrT:* N/R *Pain:*N/R	5×/week 6 weeks	Maximal cardiopulmonary test on a cyclo-ergometer at baseline and follow-up. HR_max_, VO2_max_, BP, Workload, MET, and duration-of-test were recorded.
	MICT + StrT + Stretching	*Total Exercise Duration:* 55 min *Warm-up and Cool-Down:* 5-min stationary cycling. *MICT Protocol:* 45-min continuous stationary cycling. Followed by 10-min full body (shoulder, arm, leg and hip) StrT, using 1–3 kg weights (1 × 8–10 rep), and 5-min stretching (4–5 × 20–30 s for each muscle group).	*Measurement:* HRmax (Monitorization: N/R) *Warm-Up and Cool-Down:* 50% HRmax *MICT:* 65–70% HRMax *StrT:* N/R *Pain:* N/R		
	Usual Care	Recommendations regarding exercise for fibromyalgia.	N/A		
**Flehr et al., 2019** [39]	HIIT	45-min functional training incorporating running, throwing, standing from a seated position, placing things overhead, and picking things up. *Warm-up and Demonstration:* 15 min *Movement Learning*: 15 min *HIIT Protocol:* 15-min reproduction of the movement at high intensity. Four formats possible: as fast as possible, 8-exercise tabata intervallic training followed by AerT, maximum reps or load in a set time, or as many rounds as possible in 12-min followed by AerT.	N/R *Pain*: N/R	3×/week 8 weeks	N/R
	Yoga	90-min Bikram yoga class (room at 40 °C and humidity of 40%): deep breathing, 45- to 50-min standing, stretching, and relaxation postures.	Light to moderate (According to ACSM) and sometimes vigorous. *Pain:* N/R		
**Keogh et al., 2018** [40]	HIIT	*Warm-up:* 7-min stationary cycling progressively increasing intensity. *HIIT Protocol:* 5 × 45-seg high-cadence stationary cycling alternating with 90-seg low-intensity recovery cycling. Work/Rest Ratio: [1:2] *Cool-down:* 6–7 min of light to moderate cycling.	*HIIT:* *Interval:* 110 rpm with a resistance similar or slightly higher than rest. “An intensity at which you felt it was quite difficult to complete sentences during the exercise.” *Rest:* ∼70 rpm To avoid pain, progressive increase in initial sessions.	4×/week 8 weeks	N/R
	MICT (AerT)	*Warm-up and Cool-down:* Light intensity cycling during 3 min and 2 min, respectively. *MCIT Protocol:* 20-min continuous cycling.	*MCIT:* 60–80 rpm “An intensity in which you are able to speak in complete sentences during the exercise” To avoid pain, progressive increase in initial sessions		
**Sandstad et al., 2015** [45]	HIIT	*Warm-up:* 10-min stationary cycling at moderate intensity *HIIT Protocol:* 4 × 4-min high-intensity stationary cycling alternating with 3-min cycling recovery periods. The speed and workload were adjusted continuously.	*Measurement:* HR_max_ (HR checked using HR monitor) *Warm-up:* ~70% *Interval:* 85–95% of HRmax *Rest:* ~70% of HRmax *Pain:* N/R	2×/week 10 weeks	Maximal cardiopulmonary test on a bike. VO2_max_ and HR_max_ (defined as the highest HR during the test more 5 bpm).
	Maintain daily life activities	N/A	N/A		
**Sveas et al., 2014 and 2019** [41,42]	HIIT + StrT + MICT (AerT)	Twice a week, supervised HIIT and StrT: *-HIIT Protocol:* 4 × 4-min walking/running on a treadmill alternating with 3-min of active resting. *-StrT protocol*: 20 min with external load (2–3 × 8–10 rep): Bench press or chest press machine, weighted squat or leg press machine, rowing with weight, triceps and biceps machine, and abdominal bridge. One time per week, individual interval training or MICT: 40 min of either an interval training or an MICT.	*Measurement:* HR_max_ (HR checked using HR monitor) *HIIT:* *Interval:* 90–95% HR_max_ Rest: 70% HR_max_ *MICT intensity:* >70% HRmax *Pain*: Exercises were adapted if pain reached ≥ 5/10.	3×/week 12 weeks	Cardiopulmonary test on a walking treadmill (Modified Balke protocol). VO2_max_ and HR_max_ were recorded.
	Asked to not start exercise	N/A	N/A		
**Thomsen et al., 2018 and 2019** [43,44]	HIIT	*Warm-up:* 10 min. *HIIT Protocol:* 4 × 4-min high-intensity stationary cycling alternating with a 3-min cycling recovery period. *Work/Rest Ratio:* [1:0.75] Supervised twice a week and individual once a week. Participants were instructed in using the HIIT concept by, e.g., running, bicycling, or walking uphill.	*Measurement:* HR_max_ (HR checked using HR monitor) *Interval:* 85–95% HR_max_ *Rest:* 70% HR_max_ *Pain:* N/R	2×/week 11 weeks	Maximal cardiopulmonary test on a bike. VO2_max_ and HR_max_ (defined as the highest HR during the test more 5 bpm) were recorded.
	Maintain daily physical activity	N/A	N/A		

ACSM = American College of Sports Medicine; AerT = aerobic training; bpm = beats per minute; HIIT = high-intensity interval training; HR = heart rate; HR_max_ = maximal heart rate; MICT = moderate-intensity continuous training; N/A = not applicable; rpm = revolutions per minute; StrT = strength training; VO2_max_ = maximal oxygen uptake.

**Table A3:** Assessment of the studies quality based on PEDro Scale.

Ítems												
	1	2	3	4	5	6	7	8	9	10	11	Total
Atan et al., 2020 [38]	1	1	1	1	0	0	1	1	1	1	1	**8**
Flehr et al., 2019 [39]	1	1	1	1	0	0	1	1	1	1	1	**8**
Keogh et al., 2018 [40]	1	1	1	1	0	0	0	1	1	1	1	**7**
Sandstad et al., 2015 [45]	1	1	1	1	0	0	0	1	1	1	1	**7**
Sveas et al., 2014 [42]	1	1	1	1	0	0	1	1	1	1	1	**8**
Sveas et al., 2019 [41]	1	1	1	1	0	0	1	1	1	1	1	**8**
Thomsen et al., 2018 [44]	1	1	1	1	0	0	1	1	1	1	1	**8**
Thomsen et al., 2019 [43]	1	1	1	1	0	0	1	1	1	1	1	**8**

1: subject choice criteria are specified; 2: random assignment of subjects to groups; 3: hidden assignment; 4: groups were similar at baseline; 5: all subjects were blinded; 6: all therapists were blinded; 7: all evaluators were blinded; 8: measures of at least one of the key outcomes were obtained from more than 85% of baseline subjects; 9: intention-to-treat analysis was performed; 10: results from statistical comparisons between groups were reported for at least one key outcome; 11: the study provides point and variability measures for at least one key outcome.

**Table A4:** Summary of findings and quality of evidence (GRADE).

Certainty Assessment							No. of Participants		Effect		Certainty
Outcome (No. of Studies)	Study Design	Risk of Bias	Inconsistency	Indirectness	Imprecision	Publication Bias	HIIT	Control	Relative (95% CI)	Absolute (95% CI)	
*Resting Heart Rate (5)*	RCT		Not serious	Serious	Serious	Not serious	121	127	-	−0.20 (−0.45, 0.05)	
*Resting DBP (4)*	RCT and rCOT		Not serious	Serious	Serious	Not serious	56	63	-	−0.06 (−0.43, 0.30)	
*Resting SBP (4)*	RCT and rCOT		Not serious	Serious	Serious	Not serious	56	63	-	0.07 (−0.29, 0.44)	
*Body Weight (4)*	RCT and rCOT		Not serious	Serious	Serious	Serious	89	94	-	−0.34 (−0.80, 0.12)	
*Body Fat (4)*	RCT and rCOT		Not serious	Serious	Serious	Serious	71	77	-	−0.24 (−0.57, 0.08)	
*Muscle Mass (3)*	RCT and rCOT		Not Serious	Serious	Serious	Not serious	61	63	-	0.04 (−0.32, 0.39)	
*C-Reactive Protein (4)*	RCT and rCOT		Not serious	Serious	Serious	Not serious	102	113	-	−0.05 (−0.44, 0.34)	

CI: confidence interval, DBP: diastolic blood pressure, RCT: randomized controlled trial, rCOT: randomized cross-over trial, SBP: systolic blood pressure.
